# The relationship between attachment and mental health at work: A narrative review

**DOI:** 10.1177/10519815251327313

**Published:** 2025-04-03

**Authors:** Devon McConnell, Geoff Wong, Anne Ferrey

**Affiliations:** 1 Nuffield Department of Primary Care Health Sciences, The University of Oxford, Oxford, UK

**Keywords:** stress (psychological), burnout (psychological), leadership, employee well-being, occupational health, anxiety, work performance

## Abstract

**Background:**

Well-being at work is a critical concern, with a significant portion of the workforce reporting adverse mental health impacts due to work-related stress.

**Objective:**

This narrative review explores the relationship between attachment theory and work-related mental health, focusing on how attachment influences employee experiences and outcomes.

**Methods:**

A literature search across databases, including PsycINFO, SCOPUS, and Google Scholar, was conducted, emphasizing empirical studies, systematic reviews, and meta-analyses.

**Results:**

The literature suggests that secure attachment leads to better emotional regulation, lower stress, and higher job satisfaction. Conversely, insecure attachment contributes to increased burnout, emotional distress, and lower job performance. Supportive leader-follower relationships can mitigate the adverse effects of insecure attachment by providing a secure base for employees. The review also highlights the importance of considering attachment strategies within the context of diversity and systemic challenges and recognizes that survival mechanisms in one context can be unfairly viewed as maladaptive in another.

**Conclusions:**

Enhancing secure attachment through supportive leadership and organizational practices can promote better mental health outcomes and overall workplace well-being.

## Introduction

Employers and workers alike increasingly recognize employees’ well-being as an essential concern. In a recent study of 3400 employees by The Workforce Institute, 60% of employees said work is the most significant factor in their mental health, and 69% said their manager is the most crucial factor affecting their mental health as much as their spouse. Further, 40% of senior management employees said they would quit within the year because of work-related distress.^1^ The linkage between occupational stress and the deterioration of both mental and physical health is well-established, encompassing a range of issues from anxiety and depression to migraines, sleep disturbances, and cardiovascular diseases.^2^ These mental health risks are heightened for underrepresented business leaders.^3^ Mental health problems in the workplace also affect employers, costing $187B in lost productivity in the U.S. alone – a problem intensified by the COVID-19 pandemic.^4^

Several factors influence work-related mental health, including but not limited to work-life balance, engaging tasks, communication quality, opportunities for growth, job security, and the presence of support and the ability to participate in decision-making processes.^5^ In recent years, there has been a growing scholarly interest in the role of attachment theory in treating workplace well-being.^6^ Historically, little space has been given to the topic of attachment in the workplace in organizational psychology and human resource fields.^7,8^

According to attachment theory, when faced with threats, individuals seek support from attachment figures, which is a survival mechanism.^6,9^ When there is proximity to an attachment figure who is accessible, supportive, and responsive, the individual feels secure.^10^ However, if proximity is not possible, or if the attachment figure is not supportive or responsive, the person will feel significant distress and insecurity.^10^

### Attachment theory overview

#### Origins and theory

Attachment theory, developed by John Bowlby, states that early relationships with primary caregivers shape an individual's expectations and behaviors in their later relationships. Bowlby described the attachment behavioral system as essential for survival, triggering individuals to seek support and safety from attachment figures when faced with threats.^11^ Attachment creates a felt sense of security through two mechanisms: a safe haven and a secure base. The safe haven provides comfort in times of stress, and the secure base supports autonomous exploration of one's environment.^6,12^ When people believe an attachment figure is available and can provide support in times of stress, there is a sense of felt security. They can explore the world autonomously, a phenomenon known as the dependency paradox; the more support we feel the more autonomous we can become.^6^

#### Building the internal working model of relationships

Early relationships with key caregivers help people form this internal working model of relationships they will carry with them throughout life, also called attachment styles or strategies.^6^ There are two distinct components of an attachment style, one related to the view of self and one to the view of others. The model a person forms about the self is tied to a person's view of their self-worth. The internal model of others relates to a person's belief, based on their experience, that others will be available and accessible in times of stress and need.^6,9^ These models guide emotions, cognitions, and behaviors in relationships and affect how someone responds to adversity.^10^

#### Attachment styles

The learned internal working model of relationships is expressed through patterns of behaviors that are called attachment styles. While others have been discussed and debated, the three most common attachment styles are anxious, avoidant, and secure attachment. Anxiously attached individuals worry about the availability of support and continually seek reassurance, while avoidantly attached individuals distrust support and strive for extreme self-reliance.^6,13^ Individuals with either of these insecure attachment styles will also often start using attachment strategies such as proximity seeking to be near an attachment figure or withdrawing from the attachment figure.^14,15^ In contrast, those with a secure attachment style believe others will be supportive and responsive in moments of need and, therefore show more optimism, emotional stability, and a positive view of self and others.^6,13^ Studies have shown that over half of adults classify themselves as securely attached, while the remainder are close to an even split of anxious and avoidantly attached.^16,17^

#### Secure attachment impact

People with secure attachment believe others will be there in times of need, and they trust their support, leading to greater emotional stability.^6,13^ They have more secure coping strategies in times of stress because their internal working model supports their belief that they can achieve their goals through their actions and behaviors, and they believe that others will respond positively to their actions and behaviors.^18^ Secure relationships play a critical role in a person's ability to regulate emotional well-being and social behavior.^10,11^ Secure attachment has also been linked to resiliency to stress,^14^ reduced chance of burnout,^19^ autonomy, self-efficacy, and effective emotional regulation^15,20,21^ and as a mediator of the psychological impact of traumatic events.^22^

#### Insecure attachment impact

In contrast, when an attachment figure is not accessible due to inconsistent response, loss, or another reason, it can contribute to mental health distress such as anxiety, eating disorders, suicidal ideation,^21,23^ depression and addiction,^15,24^ PTSD^21,25^ burnout,^19^ decreased emotional regulation,^26^ as well as maladaptive coping strategies, which are associated with high levels of emotional distress.^18^ Well-being conversely, increasing secure attachment in relationships can improve emotional regulation capabilities, increase resiliency, and decrease mental health distress^26^

#### Neurological basis for attachment

Attachment science has a neurological basis and a physiological impact on health. Rejection cues are processed in the same area of the brain as physical pain and in the same manner as danger.^27^ When there is a threat to security, insecure attachment develops, and the nervous system becomes hypo or hyperactive due to stress.^26,28^ This response can negatively impact cardiovascular and immune functioning and the neuroendocrine system that regulates chronic stress.^14^ Insecure attachment has also been linked to increased activity in the brain's threat response system and decreased engagement in co-regulation, making it challenging to regulate during stress.^26^

### Attachment measures

Several methods and tools are frequently used to measure adult attachment and attachment styles (see Table 1) and are included here to move from the theoretical to the practical and illustrate how attachment theory has been measured in studies included here as well as in real-world contexts.

**Table 1.** Attachment measures.

Tool	Overview	Main Usage
Adult Attachment Interview (AAI)	A semi-structured interview used to assess adult attachment representations. It builds on Bowlby's attachment theory and focuses on an individual's memories of their early attachment experiences and how these experiences influence their current functioning.^29,30^ The reliability and validity of the AAI have been extensively studied, and it has been found to be robust.^30^	Widely used in both clinical and research settings to assess attachment representations in adults, providing insights into their relational patterns and psychological well-being^30^
Experiences in Close Relationships (ECR) and ECR-Revised (ECR-R)	These self-report questionnaires are used to assess two dimensions of attachment in adult romantic relationships: anxiety and avoidance.^31,32^ The ECR-R has demonstrated high reliability and validity across diverse populations.^32^	The ECR and ECR-R are commonly used in studies examining romantic attachment and have practical applications in therapy and counselling.^32^ Recent advancements in attachment measurement include modifications to existing tools to improve their cultural sensitivity and applicability across different populations. For instance, the ECR-R has been adapted to assess attachment in non-Western cultures better.^33^
Attachment Style Questionnaire (ASQ):	This self-report questionnaire assesses adult attachment styles and has been widely used in research on attachment in adult relationships.^34^ It demonstrates good internal consistency and test-retest reliability, although its reliability may be slightly lower than the Experiences in Close Relationships (ECR) scales.^34^	While the AAI provides a deep, qualitative assessment of attachment, tools like the ECR-R and ASQ offer a more efficient quantitative approach, suitable for large-scale studies.

In summary, attachment has a broad range of impacts on a person's mental health and ability to cope with stress. Due to the central role work plays in most adults’ lives, attachment may also affect a person's ability to emotionally regulate through stressful work situations and thus may impact their work performance. Given this, attachment-related issues may have a greater impact on work-related difficulties than previously recognized. If this is the case, it may be possible to use attachment-based treatments for workplace well-being problems. This narrative review will synthesize existing literature on the relationship between attachment theory and workplace mental health, aiming to validate employees' experiences facing work-related distress and offering employers an alternative approach to supporting employee well-being.

## Methods

In this review, we took a narrative approach to synthesize articles on this topic and identify areas for future work regarding the relationship between attachment and work-related mental health. We followed the SANRA (Scale for the Assessment of Narrative Review Articles) to validate and guide the rigor of the approach and article reporting.^35^ The initial step involved identifying existing theories and literature related to attachment theory and mental health at work. Relevant literature from these searches and discussions with clinical, human resources, and academic experts in the field helped shape interpretations of the findings.

### Search strategy

**Figure 1.**
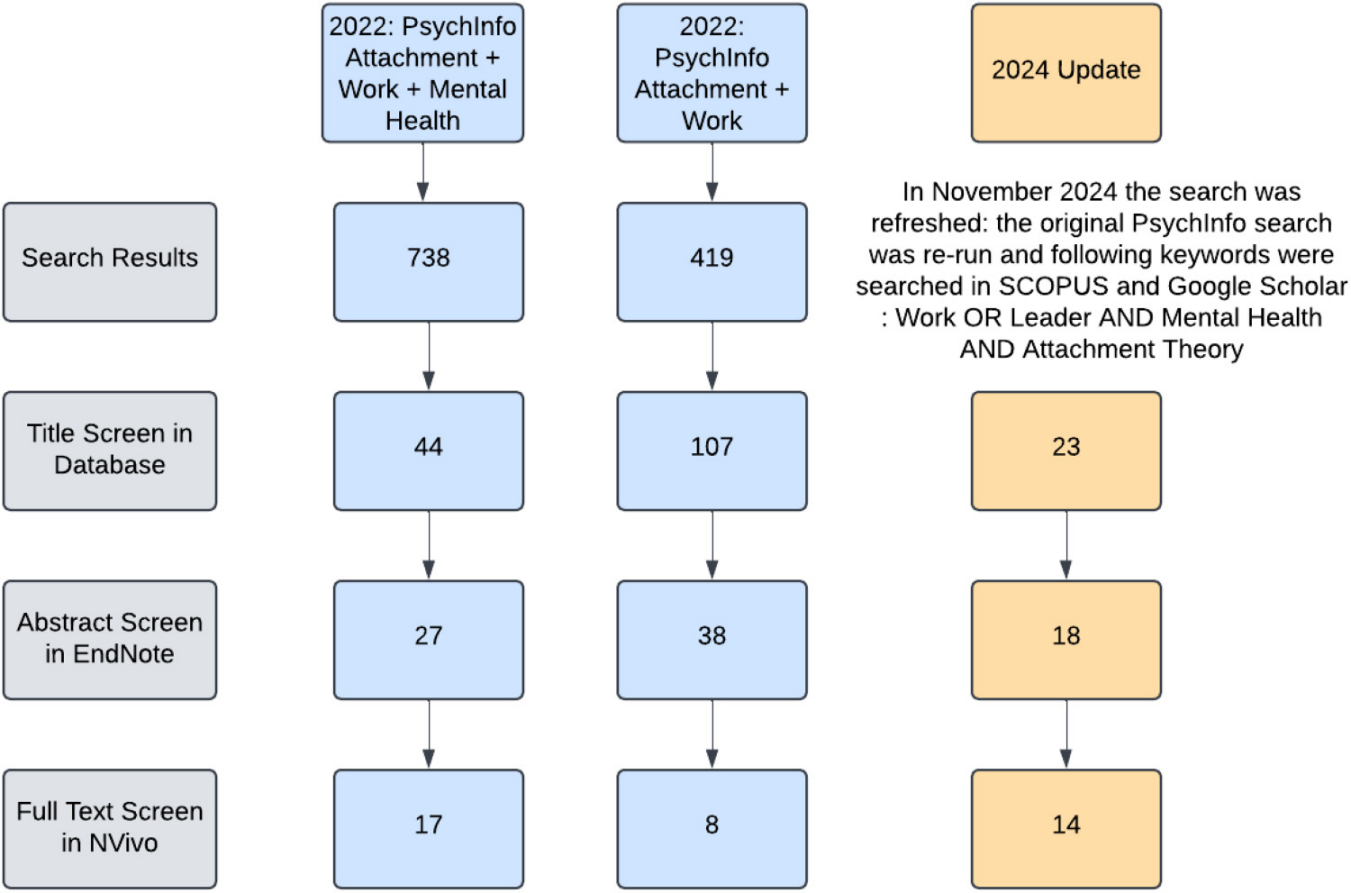
Search and article screening flow chart.

The search strategy focused on gathering data on the relationship between attachment and work-related mental health. We developed the search strategy with support from an expert librarian at the Bodleian Library at the University of Oxford. Sources included academic databases (PsycINFO, SCOPUS, and Google Scholar) and citation tracking. The initial search was conducted in November 2022, and a second search was conducted in November 2024 to update findings. Our search focused on the following keywords: “attachment AND mental health AND work,” “attachment AND work.” We used an iterative approach to refine search terms for specificity and inclusion. Additional terms to account for alternate words for mental health (e.g., anxiety) and work (e.g., business) were also included. All search results were exported to EndNote 20 for screening (Figure 1).

### Article selection

Articles included in this review were selected based on their ability to provide insights into the relationship between attachment and mental health distress in the workplace. Articles directly focused on attachment theory and work-related mental health were included and articles on attachment at work were sampled for additional context. Exclusion criteria included studies not in English, not in peer-reviewed journals, and those not involving working-age adults (Table 2). Multiple researchers reviewed a random subsample of ten percent of title and abstracts and full-text articles to ensure consistency with inclusion and exclusion criteria. Discrepancies were resolved through discussion. Citation tracking led to the inclusion of other articles that informed the narrative review.

**Figure 2.**
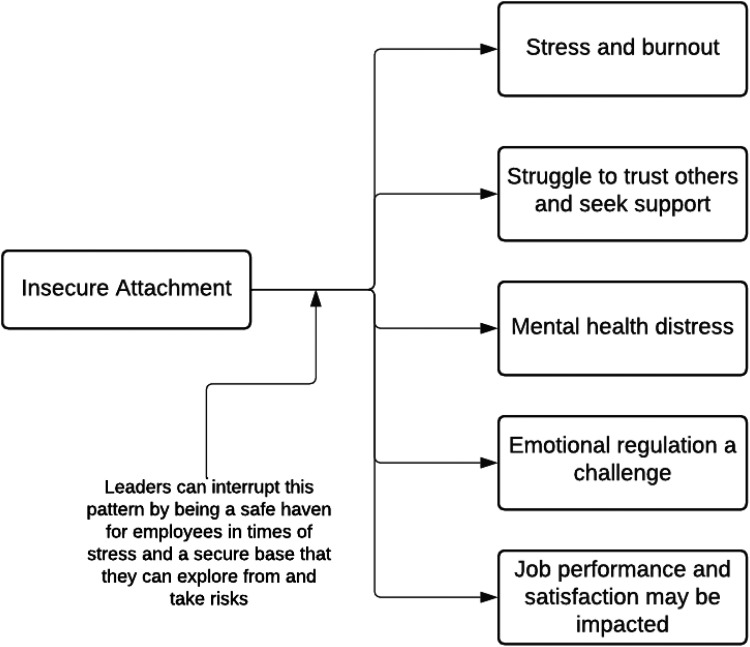
Literature indicated several potential impacts of insecure attachment.

**Table 2.** Inclusion and exclusion criteria.

Inclusion criteria	Exclusion criteria
- All articles focused on attachment theory + mental health + work, directly stated in the title or abstract - Sampled articles on attachment theory at work to provide additional background and context	Articles that were not: - In English - Published in peer-reviewed journals - With adults over age 18

### Data extraction and synthesis

All selected articles were uploaded to NVivo for coding, which included themes related to work-related distress, leadership traits, and attachment processes. Code development was inductive (from emerging themes) and deductive (based on initial theories). Broader themes were used to organize codes further. Data from the literature were synthesized to develop and refine an understanding of attachment theory's impact on work-related mental health. Judgments were made regarding the article's relevance to the research topic on the relationship between attachment and work-related mental health. These findings were iteratively refined based on ongoing stakeholder input.

## Findings

The initial search in November 2022 produced twenty-five articles that made up the core of the narrative review: seventeen related to attachment, work, and mental health, and eight additional articles more broadly on attachment at work. Articles were researched and authored in the USA (10), Israel (6), Israel & USA (2), Canada and Israel (1), Iran (1), Greece (1), Australia (1), New Zealand and USA (1), and the UK (1) and China and the USA (1). They were a range of quantitative studies (15), qualitative studies (2), commentaries (4), and reviews (4). Snowball sampling led to the inclusion of additional articles, including articles on attachment-based therapies like Emotionally Focused Individual Therapy (EFIT), which provided additional context on attachment and methods of treating insecure attachment. An additional 14 articles, 13 quantitative and one qualitative were added in November 2024, from lead researchers in Egypt (1), China (3), USA (2), Israel (2), Spain (2), Slovakia (2), France (1) and Norway (1). These additional articles further supported the findings of this narrative review that there is a relationship between attachment and mental health distress at work, including during stressful events like COVID-19.

### Attachment extends beyond the personal to the professional

Several researchers have shown that attachment models are essential factors in workplace behavior.^10,36^ According to attachment theory, emotional bonds based on care-seeking and caregiving (attachment needs) are necessary for humans to feel safe enough to explore their environment. These needs continue into adulthood through beliefs and behaviors within love and work relationships.^37^ Like personal life, work life provides an environment where exploration is required for mastery of new tasks and abilities,^36^ but this can feel difficult or even threatening. Having a secure base, meaning attachment to an available and responsive protector, can provide needed support for successful exploration.^36^ In other words, effective dependence leads to greater autonomy and self-confidence and is a hallmark of health – a counterintuitive concept known as the dependency paradox.^15^ This paradox has also been found to exist in not just our personal lives but our professional lives as well.

This narrative review synthesizes the findings on the relationships between attachment and work-related mental health. The impacts are interwoven and complex. Each person brings an internal working model of relationships shaped by their past experiences, and the dynamics at work can further evolve these models.

### Impact of attachment at work

While personal and work relationships are often considered distinct, attachment theory suggests underlying mechanisms common to both. The workplace is inherently relational, with factors such as management style and leader-follower dynamics affecting employee well-being.^8,38–40^ Humans are social beings, and for many, most waking hours are spent at work. It is therefore unsurprising that relationships and experiences at work significantly affect emotions and mental health.

Research has shown relationships between attachment and many aspects of work-related mental health and work performance, including stress and burnout; job satisfaction, performance and employee retention (Figure 2). Further, during times of significant stress like the recent COVID-19 pandemic, insecure attachment styles can increase negative mental health and physical health impacts, as was shown in a 2024 study of 1047 medical staff by Yang et al.^41^

#### Stress & burnout

Attachment systems and strategies are activated and strengthened by stress in reciprocal ways.^2^ Increased attachment insecurity has been associated with higher stress and stress symptoms.^16,38,39,42,43^ Employees who use anxious attachment strategies have also been shown to have higher stress.^42,44^ People with higher levels of attachment anxiety are significantly more likely to interpret situations as threatening and feel less confident in their ability to handle challenges, while those higher in attachment avoidance report less supportive relationships with colleagues and leaders.^2^ These findings highlight that different attachment styles influence an employee's view of themselves and others, which contribute to work-related stress. Insecure attachment has also been strongly correlated to burnout in the workplace.^8,45–48^ Burnout can be defined as emotional, physical, and cognitive exhaustion.^49^ Avoidantly attached individuals may be more likely to work long hours, prefer working alone, and take fewer holidays, putting their health and relationships at risk.^37^ It has been shown that the more securely attached an employee is, the less likely they are to burn out, and the more insecurely attached they are, the more likely they are to burn out.^16^ In a 2024 meta-analysis by Warnock et al., anxious and avoidant attachment were positively related to burnout (rc = 0.32; rc = 0.25 respectively) and secure attachment was negatively correlated (rc = -0.30).^48^

Securely attached employees build more supportive social networks at work and see that support as more positive,^2,9^ while insecurely-attached individuals may believe they cannot count on receiving support, are less capable of overcoming challenges at work, and might be more distracted when conflicts arise.^49,50^

#### Job satisfaction, performance & employee retention

Attachment security can lead to higher satisfaction in most areas of work life, including recognition, relationships with coworkers, job security, and a lower risk of experiencing psychosomatic or physical illness.^8,36^ Specifically, avoidantly attached individuals have been shown to have more negative views of organizational fairness, which has been linked to higher rates of job dissatisfaction.^45^ Insecure attachment also been significantly correlated with lower levels of innovation behaviors at work like ‘boundary spanning,’ which means collaborating and making connections with different groups to share knowledge.^51^ Further, those with insecure attachment are more likely to share an intention to leave their job.^6^

Insecure attachment is also negatively correlated to job performance.^36,48^ This can manifest as inability to complete assignments, lower satisfaction with coworker relationships, and lower performance self-ratings.^49^ When a leader is viewed as a security provider (as measured by the Leader as a Security Provider Scale – LSPS^52^) there is a statistically significant correlation to general work performance.^53^ As well, leaders contribute to their followers’ performance as attachment figures and the best scenario for an improved team performance is a secure-figure leader and followers with low separation distress, a function of secure attachment.^54^ However, work performance is nuanced and may not have as strong an impact for employees who are already highly engaged.^53^

#### Emotional regulation and surface-acting

Insecure attachment can lead to less effective emotional regulation strategies at work, like surface acting^38^ and emotional suppression.^40,55^ Surface acting, coined by Arlie Hochschild in 1983, is a form of emotional labor and occurs when workers suppress their genuine emotions, especially negative ones, to display positive affect.^38^ It has been associated with turnover, decreased well-being, increased blood pressure, and reduced social functioning and relationship formation. Employees who are concerned about social rejection may resort to surface acting as a coping strategy for anxiety, but paradoxically, this can add to their stress.^38^ Individuals who use more avoidant attachment strategies are more likely to surface act and use emotional suppression,^40^ those who use more anxious attachment strategies are more likely to emotionally ruminate and use reappraisal.^49,50^

### The impact of leaders on attachment at work

Leaders and supervisors significantly impact employees’ lives because they influence the support provided and control over one's work.^8^ Here, we use the term “leader” to encompass managers and supervisors who have some direct authority over the employee or follower. This aligns with the concept that leader-follower relationships involve attachment dynamics similar to that of a parent-child relationship.^56^ Leadership is also similar to parenting in its uneven power, the perception that a leader is stronger and wiser than the subordinate^55^ and the dual relationship between instructors and disciplinarians.^44,57^ Suppose an employee experiences stress or inconsistent leadership support. In that case, it can activate the attachment behavioral system, resulting in avoidant attachment strategies like distancing from the leader or anxious attachment strategies like proximity and attention seeking from the leader, both of which lead to counterproductive outcomes like not trusting the leader^44^ or being hypersensitive to feedback or needing excessive affirmation.^6^

One of the other hallmarks of an attachment relationship is proximity seeking during distressing times and maintaining emotional and physical availability for these moments.^9^ Leaders, like parents, can provide a secure base from which employees can feel safe to take risks and explore.^28,55,58^ As we age and view our parents as less infallible, we look to new attachment figures who are stronger and wiser such as organizational leaders like managers, religious leaders, psychotherapists, teachers, or coaches.^50,56,59^

#### Leaders provide a secure base and safe haven

Leaders are called upon to fulfil the two most important aspects of being an attachment figure: acting as a secure base and being a safe haven during times of distress.^56,58^ Responsive and supportive leaders have been shown to reduce attachment insecurity.^8,50,60^ When employees perceive more support from their supervisors, they experience less work distress and overall higher job satisfaction.^42^ Research by Wu and Parker (2017) showed that when a leader can provide this secure base, their employees show more proactive work behaviors, can take roles with more breadth, show more self-efficacy, and have more autonomous motivation.^6^ Ronen (2012) found that being an attentive, responsive, and supportive leader is a critical factor in employee job satisfaction and the prevention of burnout. Leaders who provide security (as measured by the Leader as a Security Provider Scale LSPS^61^), create a psychologically safe climate at work (which in turn was significantly related to decreasing burnout.

This has been shown to be especially true in times of stress, such as during the recent COVID-19 pandemic. During this time, leaders were sought out for comfort and support by employees regardless of attachment style or ability to work autonomously. Hinjosa, et al. (2020) recommended several strategies leaders can apply in these moments of collective stress and uncertainty to foster attachment security, including being transparent about what is known and unknown, acknowledging employee concerns, and being available for proximity seeking, including virtually.^58^

#### Impact of leader attachment styles

A leader's attachment style, meaning the attachment strategies they generally use in times of stress, impacts employees and subordinates in multiple ways. A leader's avoidant attachment style has been associated with subordinates perceiving lower cohesion among the group and having worse mental health.^55,62^ Research has also shown that the mental health of otherwise healthy and well-adapted subordinates can deteriorate when led by a leader who exhibits avoidant attachment behaviors.^25,62^ Leaders’ anxious attachment style has been associated with employees’ emotional exhaustion, lower job satisfaction, and reduced job performance.^55,63^ Leaders who showed more secure attachment were better able to support employees and their outcomes.^63^

Insecure attachment was also found to be a potential mediator in situations of authoritarian leadership; the negative impact of the authoritarian leader on a follower's sense of their value to the team was statistically less for workers with higher insecure attachment.^64^ This side of attachment theory is referred to as social deference theory, where reacting with a more urgent fight or flight response may be beneficial in certain circumstances like a fire or when under authoritarian leadership.^64^

Attachment insecurity and job performance can be mediated by an employee's level of trust in their boss.^7,19,44^ Some studies have indicated that the more an employee trusts their supervisor, the more they can focus on work tasks instead of “watching their back,” improving work performance.^19,49^ One attachment-based approach to leadership has been defined and studied, called Secure Base Leadership (SBL), which focuses on leaders exhibiting the supportive traits of providing a safe haven and secure base as discussed throughout the literature.^65^ In one study by Laguia et al. (2024) of 422 employees, SBL correlated to both task (r = 0.33, p < 0.01) and contextual (r = 0.42, p < 0.01) employee performance, with disengaged leaders having a detrimental effect on work engagement and performance.^65^

### Impact of losing a job

When someone loses their job, the impact has been shown to go beyond the financial implications to trigger mental health distress: anxiety, anger, and depression, reduced self-esteem, suicidal intentions, high blood pressure and physical illness.^10^ Research has also shown that losing one's job creates a sense of loss that is similar to feelings of abandonment after losing a critical relationship.^10^ This may be because the attachment behavioral system can be activated by interpersonal situations, such as organizational events like changes in employment.^6,10^

### Diversity and cultural considerations

Attachment is often misunderstood as personality traits, without considering the impact of systemic challenges like racism and other biases. However, when we take a systems perspective, we recognize attachment as survival strategies shaped by these and other challenges.^25^ A group's attachment style may be required for survival, promoting group functioning.^25^ All groups need people who focus on different aspects of survival: nurturing, hypervigilance against threats, avoiding fights, and similar.^25^ Individuals who often use a more anxious attachment strategy are highly perceptive and accurate detectors of threats and can navigate those dangers more effectively and quickly than those who are less anxiously attached.^25^ Fear of vulnerability is understandable for groups that have experienced ongoing biases like racism and sexism.^66^ For example, an underrepresented and oppressed group may encourage avoidant attachment strategies, like putting up a hard exterior or acting emotionless, to protect their children from abuse and discrimination. In this situation and others, avoidant behaviors are a survival response, rather than maladaptive.^25^

#### Cultural influence on attachment expectations of leaders

Many researchers view attachment science as universal due to the universal need for human connection as a basis for emotional, social, and even physical development, shown through similar patterns shown through research across various cultures.^11,22,33,63^ It also must be noted that attachment as a universal phenomenon has not been empirically proven, and behaviors viewed as insecure attachment in one culture may be viewed as secure in another.^33,66^

The idea of leadership as similar to parenthood in several key ways may not hold in every culture.^67^ In one study, which surveyed people across eight countries, there was no consistent definition of the most important leadership traits.^67^ The differences between cultures that value individualism vs. collectivism also lead to cultural differentiation in leadership and parenting. It has been shown that there is more paternalism in collectivist cultures, like Japan, which leads to employees expecting to be treated more like people instead of workers and expectations for greater job security.^67^

### Attachment styles are malleable

Attachment styles can change throughout one's lifetime through different positive or negative experiences that can create a more secure or insecure attachment style.^7,12,28^ For instance, when someone can examine contradictions within their internal models or they experience a supportive relationship with someone that matters to them, it can enable a corrective encounter.^28,67^ This experience has been called a broaden-and-build cycle, and it has been found in many dyadic settings.^50^

While attachment literature sometimes describes attachment style as a personality trait, attachment is dyadic, meaning relationship-specific, instead of one attachment style for all relationships.^6,8,44,68^ Attachment theory draws from systems theory to better understand how each person's response leads to the reaction to the other, which can lead to a problematic cycle. In a relationship, each partner may use insecure attachment strategies like proximity seeking or withdrawing to try and bring the relationship back to secure ground.^14^ When we see attachment as dyadic instead of a personality trait, it can be seen as more changeable.

#### Leaders can have an impact on an employee's pre-existing insecure attachment

Managers can be a secure base and fulfil attachment needs for acceptance and closeness, even for employees who come in with an insecure attached style^59^ which improves the employee's well-being and overall functioning.^19,49^ According to Mikulciner (2017), repeated interactions with responsive and supportive leaders can fundamentally alter a person's attachment patterns, inner working model of relationships, and psychological well-being.^37^ Mikulciner and Shaver (2016) have shown that when there is a real or symbolic supportive attachment figure present, it provides hope and optimism, increasing one's sense of self-worth and confidence in the other person's goodwill because the person feels more loved, valued, and viewed as unique.^50^ This is particularly powerful for employees with an insecure attachment style who have come to expect insensitivity and unavailability, providing them with an alternate worldview, which can change their internal working model of relationships.^67^ This effect has been found in many dyadic relationships, including teacher and student, married couples, and therapist and client.^50^ It shows that it is possible through new relationships, even at work, to create a more short-term attachment state, independent of a more general and dominant attachment style.^6^ These findings align with Bowlby's view of the plasticity of one's internal working model of relationships and attachment.^50^ Therefore, it is possible for a leader who fosters a secure attachment with an employee to make a real difference to the employee's well-being and risk of burnout.

## Discussion

This narrative review highlights the existing literature on attachment in the workplace and the possibilities inherent in using an attachment lens to understand work-related mental health. The literature suggests that when secure attachment is present, it contributes to better emotional regulation, lower stress levels, and higher job satisfaction. In contrast, insecure attachment styles are associated with an increased risk of burnout, emotional distress, and lower job performance. The workplace environment, particularly the quality of leader-follower relationships, significantly impacts employees’ mental health. Leaders who provide a secure base and act as a safe haven can mitigate the adverse effects of insecure attachment, promoting a healthier and more productive work environment.

The review also underscores the importance of considering attachment within the broader context of diversity and systemic challenges. Attachment strategies that may appear maladaptive in one context might be essential survival mechanisms in another, especially for groups facing ongoing bias and discrimination. This perspective shifts the narrative from one of individual pathology to one of resilience and adaptation. It underscores the importance of recognizing how systemic inequalities shape attachment behaviors and the necessity for therapeutic approaches that are sensitive to these broader socio-cultural dynamics. Therapists and researchers alike must consider these factors to avoid pathologizing behaviors that are adaptive responses to a challenging and often hostile environment. As Marmarosh (2022) suggests, acknowledging these interactions is vital for a more nuanced understanding of attachment and its implications for mental health, particularly in diverse populations.

The literature underscores that attachment is a malleable internal model, not a fixed personality trait. This suggests that regardless of whether an employee enters the workplace with a secure or insecure attachment style shaped by their childhood and past relationships, their work relationships—especially with their leader—can significantly influence and reshape these internal models. For instance, an employee with an insecure attachment, rooted in early life experiences, may encounter a leader who offers a secure base and a safe haven during stressful moments. This new relational experience can transform the employee's beliefs about their environment, leading to shifts in their attachment strategies and behaviors. Conversely, an employee who begins with a secure attachment may, when exposed to a neglectful or abusive leader, begin to exhibit insecure attachment strategies such as avoidance or proximity-seeking. These findings highlight the critical role supportive leadership plays in employee performance and mental health and challenge the notion that attachment styles are fixed and unchangeable, regardless of early life experiences.

### Strengths, limitations & future research directions

One of the key strengths of this narrative review lies in its detailed synthesis of existing literature on the relationship between attachment theory and mental health in the workplace. By integrating findings from diverse studies, this review provides a nuanced understanding of how attachment styles influence employee well-being and organizational outcomes. Additionally, the review highlights the critical role of leadership in moderating these effects, offering practical implications for enhancing workplace mental health through supportive leader-follower relationships. However, several limitations must be acknowledged. Firstly, the narrative nature of the review may introduce selection bias, as the included studies were chosen based the authors’ determination of their relevance as well as availability. Secondly, while the review incorporates studies from various cultural contexts, the generalizability of the findings may be limited due to cultural differences in attachment behaviors and workplace dynamics. Also, there are many additional variables beyond attachment that the literature does not disentangle, like job functions and levels, industries, personalities, education and others. Future studies should consider longitudinal designs that track the evolution of attachment styles in employees over time and across different cultural contexts to provide a more comprehensive understanding of workplace dynamics. Additional research can also focus on potential attachment-based treatment options for work-related mental health distress, the impact of co-workers on attachment at work, as well as a deeper examination of how culture influences attachment in various workplace environments.

### Implications for policy

The findings of this narrative review could have significant implications for workplace policies aimed at improving mental health and well-being. Organizations may consider further fostering supportive leader-follower relationships, which have been shown to mitigate the adverse effects of insecure attachment and enhance overall job satisfaction and performance. Training programs and coaching for managers and leaders could be implemented to help them understand the importance of acting as a secure base and a safe haven for employees, providing both support and opportunities for professional growth. Therapists and coaches could also receive training on the link between attachment and work-related mental health as well as attachment-based tools and treatment options. By integrating these attachment-based approaches into organizational policies and practices, employers are more likely to create a healthier, more supportive work environment that enhances employee well-being, reduces turnover, and improves productivity.

## Conclusion

Attachment theory offers a valuable framework for understanding and addressing work-related mental health issues. Secure attachment has numerous positive outcomes, including better emotional regulation, reduced stress, and higher job satisfaction. Conversely, insecure attachment can lead to significant mental health challenges, affecting both individual well-being and organizational performance.

Leaders play a pivotal role in shaping the attachment dynamics within the workplace. By fostering secure attachments, they can create an environment that supports mental health and enhances overall productivity. Future research should continue to explore the complex interplay between attachment theory, mental health, and workplace dynamics, with particular attention to the diverse experiences of different employee groups and potential attachment-based treatment options.
